# Second primary malignancies in colorectal cancer patients

**DOI:** 10.1038/s41598-021-82248-7

**Published:** 2021-02-02

**Authors:** Jana Halamkova, Tomas Kazda, Lucie Pehalova, Roman Gonec, Sarka Kozakova, Lucia Bohovicova, Dagmar Adamkova Krakorova, Ondrej Slaby, Regina Demlova, Marek Svoboda, Igor Kiss

**Affiliations:** 1 Department of Comprehensive Cancer Care, Masaryk Memorial Cancer Institute, Brno, Czech Republic; 2 Department of Comprehensive Cancer Care, Faculty of Medicine, Masaryk University, Brno, Czech Republic; 3 Department of Medical Ethics, Faculty of Medicine, Masaryk University, Brno, Czech Republic; 4 Department of Radiation Oncology, Masaryk Memorial Cancer Institute, Zluty kopec 7, Brno, 625 00 Czech Republic; 5 Department of Radiation Oncology, Faculty of Medicine, Masaryk University, Brno, Czech Republic; 6 Institute of Health Information and Statistics of the Czech Republic, Prague, Czech Republic; 7 Institute of Biostatistics and Analyses, Faculty of Medicine, Masaryk University, Brno, Czech Republic; 8 Department of Pharmacy, Masaryk Memorial Cancer Institute, Brno, Czech Republic; 9 Department of Pharmaceutics, Faculty of Pharmacy, Masaryk University, Brno, Czech Republic; 10 Central European Institute of Technology, Brno, Czech Republic; 11 Department of Pharmacology, Faculty of Medicine, Masaryk University, Brno, Czech Republic; 12 Clinical Trial Unit, Masaryk Memorial Cancer Institute, Brno, Czech Republic

**Keywords:** Cancer epidemiology, Gastrointestinal cancer

## Abstract

The prevalence of second primary malignancies (SPMs) in the western world is continually increasing with the risk of a new primary cancer in patients with previously diagnosed carcinoma at about 20%. The aim of this retrospective analysis is to identify SPMs in colorectal cancer patients in a single-institution cohort, describe the most frequent SPMs in colorectal cancer patients, and discover the time period to occurrence of second primary tumors. We identified 1174 patients diagnosed with colorectal cancer in the period 2003–2013, with follow-up till 31.12.2018, and median follow-up of 10.1 years, (median age 63 years, 724 men). A second primary neoplasm was diagnosed in 234 patients (19.9%). Older age patients, those with early-stage disease and those with no relapse have a higher risk of secondary cancer development. The median time from cancer diagnosis to development of CRC was 8.9 years for breast cancer and 3.4 years for prostate cancer. For the most common cancer diagnosis after primary CRC, the median time to development was 0–5.2 years, depending on the type of malignancy. Patients with a diagnosis of breast, prostate, or kidney cancer, or melanoma should be regularly screened for CRC. CRC patients should also be screened for additional CRC as well as cancers of the breast, prostate, kidney, and bladder. The screening of cancer patients for the most frequent malignancies along with systematic patient education in this field should be the standard of surveillance for colorectal cancer patients.

## Introduction

Colorectal cancer (CRC) is the second most common cause of cancer death in the United States. It is estimated that in 2020 there will be 147,950 patients diagnosed and 53,200 will die from the disease^1^. The prevalence of second primary malignancies in the western world is continually increasing^2^. Due to screening programs and the success of personalized therapy, the mortality rate from this disease has decreased. In 2015, CRC prevalence in the Czech Republic (population 10.5 mil in 2015) reached 64,126 patients (6107/mil inhabitants). This is in comparison with 2005 (population 10.2 mil in 2005), there were 46,053 patients (4515/mil inhabitants) which was an increase of almost 40%^3^.

As the survival rates of cancer patients improves, those patients are more likely to develop SPMs. The type of SPMs and the frequency of occurrence is important in the field of cancer surveillance. The risk of a new primary cancer in patients with previously diagnosed carcinoma is about 20%, and more than one other cancer is diagnosed in approximately 30% of cancer survivors aged > 60 years^4,5^. As the number of cancer survivors increases, the occurrence of multiple primary cancers is also likely to rise. The most common subsequent cancers in the western world are nonmelanoma skin cancer, colorectal cancer, and breast cancer^2^. Primary and secondary malignancies are associated with lifestyle, environmental risk factors, host factors and hereditary susceptibility^6^. In secondary tumors, the late toxicity from previous anticancer therapy is also significant. Patients with SPMs after primary CRC have a worse prognosis than those with only a CRC diagnosis^7^.

Follow-up of CRC survivors was developed on the basis of limited resources, irrespective of the higher incidence of secondary malignancies. High-quality surveillance with the determination of duration, frequency, and method for the screening of SPMs is still missing.

The aim of this retrospective analysis is to identify SPMs in colorectal cancer patients in a single-institution cohort, describe the most frequent SPMs in colorectal cancer patients, and discover the time period to occurrence of second primary tumors.

## Material and methods

### Patient selection

After approval by the ethics committee (number 2019/1827/MOU), patients with CRC diagnosed in the period 2003–2013 and followed-up by the end of 2018 at the Masaryk Memorial Cancer Institute (MMCI) in Brno, Czech Republic, were screened for eligibility after gaining their written informed consent for dealing with personal data in regard to the research. Data of those meeting the following criteria were retrieved from electronic medical records: adult patients aged ≥ 18 years with a CRC diagnosis confirmed by positive histology. Exclusion criteria were: (1) CRC diagnosed at autopsy, (2) patients lost to follow-up, (3) patients with a high risk of development SPMs due to hereditary cancer syndrome (e.g. BRCA 1, 2, Lynch syndrome, familial adenomatous polyposis (FAP)). We included cases of carcinoma in situ and clinically localized, regionally advanced, and metastatic disease.

### Second primary malignancies

Multiple primaries are defined as more than one synchronous or metachronous cancer in the same individual. For epidemiological studies, tumors are considered multiple primary malignancies if they arise in different sites and/or are of a different histology or morphological group^8^. For the definition of site of the tumor in our study, criteria according to the SEER definition of multiple primary tumors was used, i.e. multiple primaries are: (1) tumors with ICD-O-3 histology codes that are different to the first, second or third number; (2) tumors with ICD-O-3 topography codes that are different at the second and/or third characters^9^.

Synchronicity was qualified according to the rules of the International Agency for Research on Cancer (IARC) which suggest the registration of synchronous tumours diagnosed in an interval of fewer than 6 months (or metachronous if more than 6 months) if arising in different sites^10^.

### Statistical analysis

Comparisons of basic characteristics between the patients with SPM and the patients without SPM were summarized with counts and frequencies and tested with the Fisher exact test and Mann–Whitney test in case of categorical characteristics and continuous characteristics, respectively. Considering the sidedness of CRC, the International Classification of Diseases for Oncology coding scheme was used to categorize by the primary site as either: right colon (cecum, ascending colon, hepatic flexure), left colon (splenic flexure, descending colon, sigmoid colon), or rectum (rectosigmoid, rectum). The transverse colon (C18.4) was excluded from the laterality assessment (45 patients), as it was only possible by ICD-O-3 topography codes to define assignment to the right or left colon.

Logistic regression models were used to determine predictors of occurrence of SPM in patients with CRC. The following covariates were examined: gender, age at CRC diagnosis, clinical stage, status of relapse and sidedness of CRC. Grade and KRAS status were not examined due to the large number of missing records. Patients with an unknown clinical stage and a diagnosis of transverse colon (C18.4) were removed from the analysis (91 patients). Each covariate was fit univariately in separate logistic regression models. One overall multivariate logistic model including all covariates was used to assess independent effects.

Occurrences of SPMs by the site of diagnosis were described by counts and frequencies. SPMs with an unknown date of diagnosis were not included in this analysis (7 SPMs). The national cancer registry of the Czech Republic (NOR)^11^ was used to compare the frequencies of sites of diagnosis in our study with the frequencies in the entire Czech population.

The time from the diagnosis of previous neoplasm to the diagnosis of the first colorectal cancer and the time from the diagnosis of the first colorectal cancer to the diagnosis of subsequent neoplasm were described by mean and median. SPMs with an unknown date of diagnosis were not included in this analysis (7 SPMs).

Kaplan‐Meier curves were utilized to display the survival of the patients with colorectal cancer stratified by the occurrence of an SPM. The primary endpoint used was 15-year survival. Observations with 15 or more years of follow-up were censored at 15 years. The Breslow test was used to compare the differences in survival between defined groups of patients with respect to the occurrence of a SPM. The hazard ratio (HR) with corresponding 95% confidence interval was determined based on the Cox proportional hazards model, adjusted to gender, age at diagnosis, clinical stage, status of relapse and sidedness of CRC. The relationship between the occurrence of SPMs and presence of radiotherapy/chemotherapy was tested by the Fisher exact test.

### Ethics approval

We confirm that this manuscript has not been published elsewhere and is not under consideration by another journal. All authors have contributed significantly and are in agreement with the content of the manuscript. This retrospective chart review study involving human participants was in accordance with the ethical standards of the institutional and national research committee and with the 1964 Helsinki Declaration and its later amendments or comparable ethical standards. The Institutional review Board of Masaryk Memorial Cancer Institute approved this study (number 2019/1827/MOU).

## Results

### Patients selection

In total, 1174 CRC patients diagnosed in the period from 1.1.2003 till 31.12.2013 were identified and enrolled in this study. Follow-up was continued till 31.12.2018, with median follow-up of 10.1 years, (median age 63 years, 724 men). The baseline patient characteristics are summarized in Table 1.

**Table 1:** Characteristics of colorectal cancer patients (C18–C20) stratified by occurrence of second primary malignancies.

	No SPM (N = 940) Count (%)	With SPM (N = 234) Count (%)	*p* value
**Gender**			
Men	590 (62.8)	134 (57.3)	0.133^1^
Women	350 (37.2)	100 (42.7)	
**Age at CRC diagnosis**			
0–44	79 (8.4)	14 (6.0)	**0.001**^1^
45–54	153 (16.3)	21 (9.0)	
55–64	296 (31.5)	58 (24.8)	
65–74	278 (29.6)	93 (39.7)	
75+	134 (14.3)	48 (20.5)	
Median (5%–95% percentile)	63 (55–70)	67 (60–73)	**< 0.001**^2^
**Clinical stage**			
Complete records	906 (96.4)	221 (94.4)	**0.012**^1^
Stage I + in situ	249 (27.5)	68 (30.8)	
Stage II	218 (24.1)	67 (30.3)	
Stage III	260 (28.7)	61 (27.6)	
Stage IV	179 (19.8)	25 (11.3)	
Not available	34 (3.6)	13 (5.6)	
**Grade**			
Complete records	616 (65.5)	180 (76.9)	0.464^1^
1	162 (26.3)	45 (25.0)	
2	344 (55.8)	109 (60.6)	
3	110 (17.9)	26 (14.4)	
Not available	324 (34.5)	54 (23.1)	
**Relapse**			
Yes	314 (33.4)	44 (18.8)	**< 0.001**^1^
No	626 (66.6)	190 (81.2)	
**KRAS**			
Complete records	222 (23.6)	28 (12.0)	0.418^1^
Positive	91 (41.0)	9 (32.1)	
Negative	131 (59.0)	19 (67.9)	
Not available	717 (76.3)	176 (75.2)	

^1^Fischer exact test.

^2^Mann–Whitney test. SPM, second primary malignancy; CRC, colorectal cancer.

^3^K ras status was performed in the surgical specimen in non-metastatic patients or in the case of surgical treatment and biopsy specimen in metastatic patients without any surgery.

^4^The transverse colon (C18.4) was excluded from the laterality assessment, as it was only possible by ICD-O-3 topography codes to define assignment to the right or left colon (45 patients).

### Second primary malignancies

We did not find any statistically significant difference between patients with and without an SPM with respect to gender, the grade of the tumor, or KRAS status. However, for age at diagnosis, clinical stage, and status of relapse significant differences were revealed (Table 1). Considering sidedness of CRC, it was evident that patients with rectal cancer are less likely to have SPMs than patients with colon cancer, however, the *p* value for sidedness did not reach statistical significance. NRAS as well as BRAF status was not assessed as this information was missed in the majority of patients due to the evaluated time period.

Based on univariate logistic models it was shown that patients aged 65 and over are approximately two times more likely to develop SPM than patients under 45 years (Table 2). Similarly, increased odds were detected in patients without relapse. In contrast, a significantly lower chance of SPM was demonstrated for clinical stage IV compared to stage I (OR = 0.49, *p* = 0.006) and localization in rectum compared to right colon (OR = 0.66, *p* = 0.039). In the multivariate model, after considering the effect of all variables analyzed, a statistically increased chance of SPM was reported only for patients without relapse (OR = 1.79; *p* = 0.004). Age at diagnosis over 65 years and clinical stage IV did not reach statistical significance in the multivariate model, however, the detected OR values were still very different from 1.

**Table 2:** Odds ratios for occurrence of second primary malignancies derived from the logistic regression models (N = 1083).

	Univariate		Multivariate	
	OR (95% CI)	*p* value	OR (95% CI)	*p* value
**Gender**				
Men	1.00		1.00	
Women	1.30 (0.96–1.76)	0.097	1.21 (0.88–1.65)	0.243
**Age at CRC diagnosis**				
0–44	1.00		1.00	
45–54	0.86 (0.40–1.85)	0.695	0.89 (0.41–1.94)	0.768
55–64	1.14 (0.58–2.25)	0.708	1.13 (0.57–2.25)	0.720
65–74	1.89 (0.98–3.66)	0.058	1.80 (0.92–3.50)	0.086
75+	2.14 (1.07–4.31)	**0.033**	1.80 (0.88–3.66)	0.106
**Clinical stage**				
Stage I + in situ	1.00		1.00	
Stage II	1.07 (0.72–1.58)	0.749	0.99 (0.66–1.49)	0.968
Stage III	0.81 (0.55–1.20)	0.298	0.86 (0.57–1.31)	0.488
Stage IV	0.49 (0.30–0.82)	**0.006**	0.63 (0.36–1.09)	0.096
**Relapse**				
Yes	1.00		1.00	
No	2.20 (1.52–3.18)	**< 0.001**	1.79 (1.20–2.67)	**0.004**
**Laterality**				
Right colon (C18.0–C18.3)	1.00		1.00	
Left colon (C18.5–C19)	0.81 (0.53–1.24)	0.328	0.98 (0.63–1.51)	0.921
Rectum (C20)	0.66 (0.45–0.98)	**0.039**	0.77 (0.51–1.17)	0.219

CRC, colorectal cancer; OR, odds ratio; CI, confidence interval.

Patients with an unknown clinical stage and a diagnosis of transverse colon (C18.4) were removed from the analysis (91 patients).

**Figure 1:**
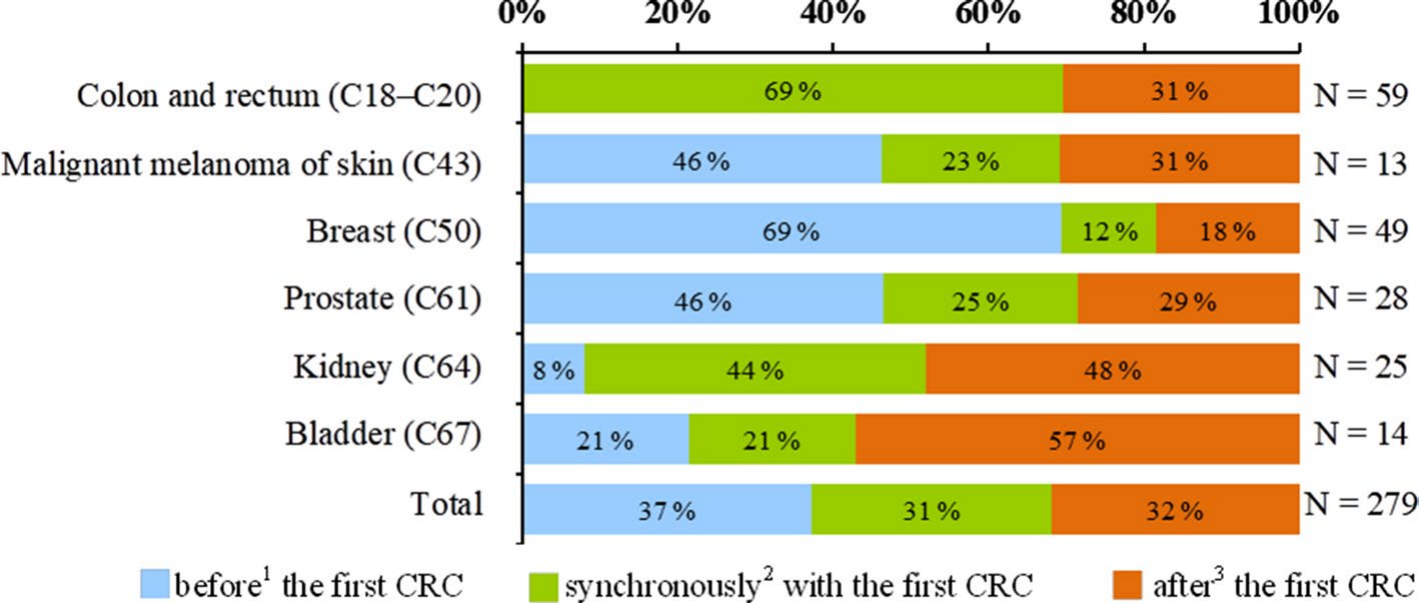
Occurrence of second primary malignancies by the time of diagnosis. Only SPMs with known date of diagnosis were considered (date of diagnosis was not available for 7 SPMs). Only sites of diagnosis with total SPMs greater than 10 are shown. ^1^Diagnosed 6 or more months before the first CRC in the patient. ^2^Diagnosed within 6 months before or after the first CRC in the patient. ^3^Diagnosed 6 or more months after the first CRC in the patient. SPM, multiple primary neoplasm; CRC, colorectal cancer.

A second primary neoplasm was diagnosed in 234 patients (Table 3), one secondary neoplasm was found overall in 190 (16.2%), 36 (3.1%) patients suffered from two SPMs, and 8 (0.7%) were treated with three SPMs. Among SPMs, colorectal cancer (21.1%), breast cancer (17.6%) and prostate cancer (10.0%) were the most represented diagnoses (Table 4). Considering the relatively high proportion of men (62%) in our study, the incidence of prostate cancer is only slightly increased compared to general population, while the incidence of breast cancer is even more pronounced and indicates a significant risk of CRC occurance. The description of the occurrence of multiple primary neoplasms diagnosed before, synchronously, or after diagnosis of CRC is listed in Table 3. The majority of breast cancer and almost half of melanoma cases preceded the CRC diagnosis as well as a diagnosis of prostate cancer, where the distribution of patients over time is more homogenous. Renal cancer was diagnosed predominantly synchronously and after CRC diagnosis, as well as bladder cancer, where two-thirds of cases were diagnosed after CRC (Table 4; Fig. 1). The total number of secondary tumors was homogeneously stratified over time, approximately one third of the secondary tumors were diagnosed before, 1/3 synchronously, and 1/3 after the diagnosis of CRC. Table 5 summarizes the time from the previous neoplasm to the diagnosis of the SPMs. The median time from diagnosis to the development of CRC is 8.9 years for breast cancer and 3.4 years for prostate cancer. The time to second colorectal cancer is shown only for the site of diagnosis with the number of previous primary neoplasms greater than 10.

**Table 3:** Occurrence of second primary malignancy with respect to the first colorectal cancer (C18–C20) in the patient. Number of secondary neoplasm is presented as well.

	Men		Women		Total	
	Number of patients (%) (N = 724)	Number of SPM (N = 160)	Number of patients (%) (N = 450)	Number of SPM (N = 126)	Number of patients (%) (N = 1174)	Number of SPM (N = 286)
No SPM	590 (81.5)	–	350 (77.8)	–	940 (80.1)	–
With SPM	134 (18.5)	160	100 (22.2)	126	234 (19.9)	286
Before^1^ the first CRC	40 (5.5)	44	54 (12.0)	60	94 (8.0)	104
Synchronously^2^ with the first CRC	52 (7.2)	58	27 (6.0)	28	79 (6.7)	86
After^3^ the first CRC	48 (6.6)	51	35 (7.8)	38	83 (7.1)	89
Date of SPM diagnosis NA	6 (0.8)	7	0 (0.0)	0	6 (0.5)	7
With SPM	134 (18.5)		100 (22.2)		234 (19.9)	
One secondary neoplasm	112 (15.5)		78 (17.3)		190 (16.2)	
Two secondary neoplasms	18 (2.5)		18 (4.0)		36 (3.1)	
Three secondary neoplasms	4 (0.6)		4 (0.9)		8 (0.7)	

^1^Diagnosed 6 or more months before the first CRC in the patient.

^2^Diagnosed within 6 months before or after the first CRC in the patient.

^3^Diagnosed 6 or more months after the first CRC in the patient.

SPM, second primary malignancy; CRC, colorectal cancer; NA, not available.

**Table 4:** Second primary malignancies by the site of diagnosis.

	SPM before^1^ the first CRC (%) (N = 104)	SPM synchronously^2^ with the first CRC (%) (N = 86)	SPM after^3^ the first CRC (%) (N = 89)	Total SPM (%) (N = 279)	All malignant neoplasms according to NOR (%) (N = 2 367 973)
Oral cavity and pharynx (C00–C14)	3 (2.9)	1 (1.2)	3 (3.4)	7 (2.5)	47 097 (2.0)
Esophagus (C15)	0 (0.0)	0 (0.0)	1 (1.1)	1 (0.4)	16 943 (0.7)
Stomach (C16)	1 (1.0)	2 (2.3)	3 (3.4)	6 (2.2)	84 738 (3.6)
Colon and rectum (C18–C20)	0 (0.0)	41 (47.7)	18 (20.2)	59 (21.1)	268 753 (11.3)
Liver and intrahepatic bile ducts (C22)	0 (0.0)	2 (2.3)	2 (2.2)	4 (1.4)	30 775 (1.3)
Gallbladder and biliary tract (C23, C24)	0 (0.0)	0 (0.0)	0 (0.0)	0 (0.0)	39 697 (1.7)
Pancreas (C25)	1 (1.0)	1 (1.2)	0 (0.0)	2 (0.7)	65 789 (2.8)
Larynx (C32)	2 (1.9)	0 (0.0)	0 (0.0)	2 (0.7)	21 055 (0.9)
Lung, bronchus and trachea (C33, C34)	2 (1.9)	2 (2.3)	5 (5.6)	9 (3.2)	249 926 (10.6)
Malignant melanoma of skin (C43)	6 (5.8)	3 (3.5)	4 (4.5)	13 (4.7)	56 372 (2.4)
Other malignant neoplasms of skin (C44)	2 (1.9)	3 (3.5)	2 (2.2)	7 (2.5)	532 199 (22.5)
Soft tissues (C47, C49)	1 (1.0)	0 (0.0)	0 (0.0)	1 (0.4)	10 358 (0.4)
Breast (C50)	34 (32.7)	6 (7.0)	9 (10.1)	49 (17.6)	199 562 (8.4)
Cervix uteri (C53)	6 (5.8)	1 (1.2)	0 (0.0)	7 (2.5)	43 373 (1.8)
Uterus (C54, C55)	5 (4.8)	0 (0.0)	1 (1.1)	6 (2.2)	66 192 (2.8)
Ovary (C56)	1 (1.0)	0 (0.0)	4 (4.5)	5 (1.8)	42 593 (1.8)
Prostate (C61)	13 (12.5)	7 (8.1)	8 (9.0)	28 (10.0)	142 994 (6.0)
Testis (C62)	4 (3.8)	0 (0.0)	0 (0.0)	4 (1.4)	14 440 (0.6)
Kidney (C64)	2 (1.9)	11 (12.8)	12 (13.5)	25 (9.0)	85 270 (3.6)
Bladder (C67)	3 (2.9)	3 (3.5)	8 (9.0)	14 (5.0)	69 826 (2.9)
Central nervous system (C70–C72)	0 (0.0)	0 (0.0)	0 (0.0)	0 (0.0)	27 516 (1.2)
Thyroid gland (C73)	1 (1.0)	0 (0.0)	3 (3.4)	4 (1.4)	23 545 (1.0)
Hodgkin's disease (C81)	1 (1.0)	0 (0.0)	0 (0.0)	1 (0.4)	12 082 (0.5)
Non-Hodgkin's lymphoma (C82–C86)	5 (4.8)	1 (1.2)	0 (0.0)	6 (2.2)	41 122 (1.7)
Multiple myeloma (C90)	1 (1.0)	0 (0.0)	0 (0.0)	1 (0.4)	17 252 (0.7)
Leukemia (C91–C95)	3 (2.9)	0 (0.0)	3 (3.4)	6 (2.2)	46 717 (2.0)
Other malignant neoplasms	7 (6.7)	2 (2.3)	3 (3.4)	12 (4.3)	111 787 (4.7)

Only SPMs with known date of diagnosis were considered (date of diagnosis was not available for 7 SPMs).

^1^Diagnosed 6 or more months before the first CRC in the patient.

^2^Diagnosed within 6 months before or after the first CRC in the patient.^3^ diagnosed 6 or more months after the first CRC in the patient.

SPM, second primary malignancy; CRC, colorectal cancer, NOR, national cancer registry (1977–2017).

**Table 5:** Previous neoplasms before second colorectal cancer. Second primary malignancy after colorectal cancer diagnosis.

	Number of previous^1^ primary neoplasms (%)	Time from the previous^1^ neoplasm to the diagnosis of the first CRC		Number of second^2^ primary malignancy (%)	Time from the first CRC diagnosis to second^2^ SPM	
		Mean (years)	Median (years)		Mean (years)	Median (years)
Oral cavity and pharynx (C00–C14)	4 (3.0)	–	–	3 (2.1)	–	–
Stomach (C16)	3 (2.2)	–	–	3 (2.1)	–	–
Esophagus (C15)	–	–	–	1 (0.7)	–	–
Colon and rectum (C18–C20)	–	–	–	59 (41.0)	1.7	0.0
Liver and intrahepatic bile ducts (C22)	2 (1.5)	–	–	2 (1.4)	–	–
Pancreas (C25)	2 (1.5)	–	–	–	–	–
Larynx (C32)	2 (1.5)	–	–	–	–	–
Lung, bronchus and trachea (C33, C34)	3 (2.2)	–	–	6 (4.2)	–	–
Malignant melanoma of skin (C43)	9 (6.7)	–	–	4 (2.8)	–	–
Other malignant neoplasms of skin (C44)	5 (3.7)	–	–	2 (1.4)	–	–
Soft tissues (C47, C49)	1 (0.7)	–	–			
Breast (C50)	37 (27.4)	8.4	8.9	12 (8.3)	4.7	4.4
Cervix uteri (C53)	7 (5.2)	–	–	–	–	–
Uterus (C54, C55)	5 (3.7)	–	–	1 (0.7)	–	–
Ovary (C56)	1 (0.7)	–	–	4 (2.8)	–	–
Prostate (C61)	16 (11.9)	5.7	3.4	12 (8.3)	3.9	5.2
Testis (C62)	4 (3.0)	–	–	–		
Kidney (C64)	9 (6.7)	–	–	16 (11.1)	3.9	2.0
Bladder (C67)	4 (3.0)	–	–	10 (6.9)	2.8	2.5
Thyroid gland (C73)	1 (0.7)	–	–	3 (2.1)	–	–
Hodgkin's disease (C81)	1 (0.7)	–	–	–	–	–
Non-Hodgkin's lymphoma (C82–C86)	6 (4.4)	–	–	–	–	–
Multiple myeloma (C90)	1 (0.7)	–	–	–	–	–
Leukemia (C91–C95)	3 (2.2)	–	–	3 (2.1)	–	–
Other malignant neoplasms	9 (6.7)	–	–	3 (2.1)	–	–
Total	135 (100.0)	7.3	3.3	144 (100.0)	3.1	1.4

Only neoplasms with known date of diagnosis were considered. Time to second colorectal cancer is shown only for site of diagnosis with number of previous primary neoplasms greater than 10.

^1^Diagnosed before the first CRC in the patient (both synchronous (within 6 months before the first CRC) and metachronous (more than 6 months before the first CRC) second neoplasms were considered).

CRC, colorectal cancer.

^2^Diagnosed the same day or after the first CRC (both synchronous (within 6 months after the first CRC) and metachronous (more than 6 months after the first CRC) second neoplasms were considered).

SPM, second primary malignancy; CRC, colorectal cancer.

As indicated in Table 5, the most common cancer diagnosis was found to be a median of 0–5.2 years after primary CRC. As well as the previous, the time to subsequent colorectal cancer is shown only for the site of diagnosis with the number of subsequent primary neoplasms greater than 10, for the reason of possible bias using small numbers of patients in a particular diagnosis.

**Figure 2:**
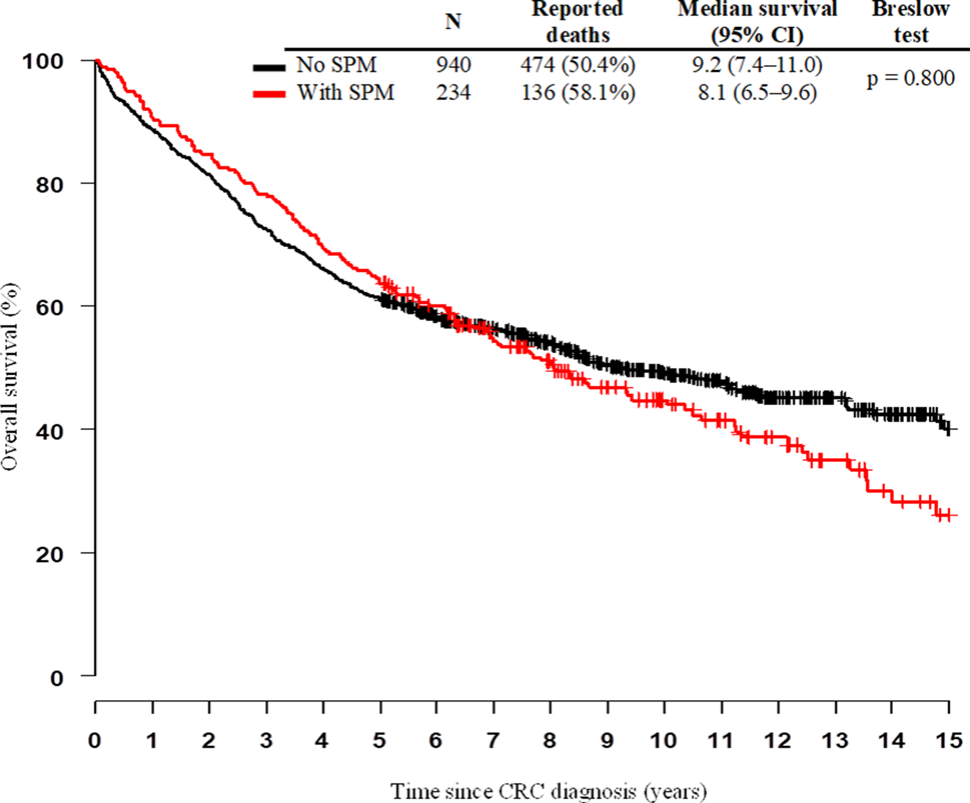
Kaplan–Meier curves of 15-year survival among colorectal cancer patients (C18–C20) stratified by occurrence of second primary malignancy. SPM, second primary malignancy; CRC, colorectal cancer, CI, confidence interval.

**Figure 3:**
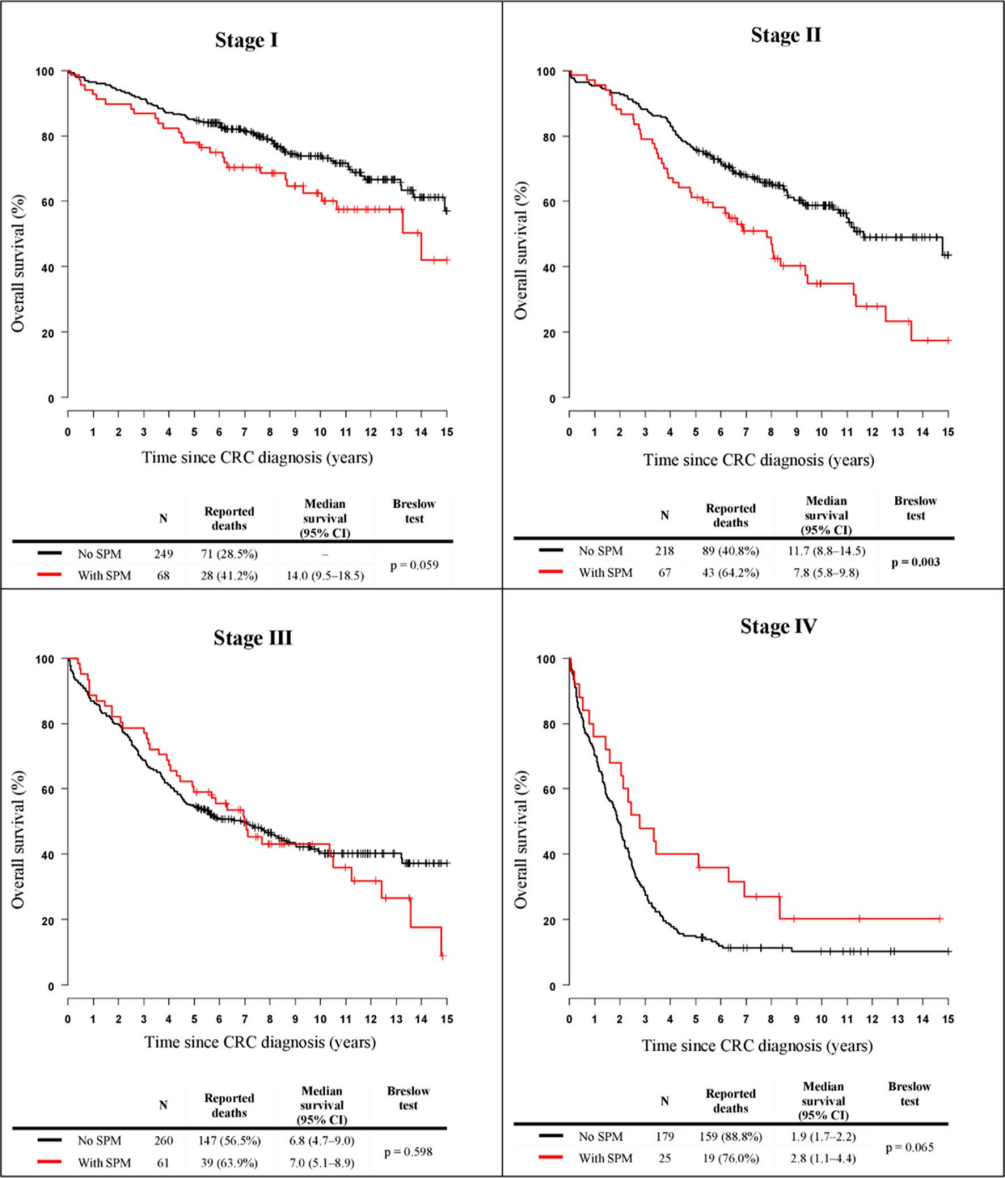
Kaplan–Meier curves of 15-year survival among colorectal cancer patients (C18–C20) stratified by occurrence of second primary malignancy, depending on the stage of the disease. SPM, second primary malignancy; CRC, colorectal cancer, CI, confidence interval.

The Kaplan–Meier curves of 15-year survival among colorectal cancer patients stratified by the occurrence of multiple primary neoplasms show better OS for patients with SPMs in the first 6 years and therefore OS was lower (Fig. 2), but this difference is not statistically significant. The differences in the clinical stages are shown in Fig. 3. Patients with SPM showed significantly worse survival in earlier clinical stages (stages I and II) compared with patients without SPM. In contrast, in advanced metastatic disease (grade IV), patients with SPM showed better survival than patients without SPM. In stage III, survival was comparable between the two groups of patients. Patients with early stages of CRC stay alive longer and have a greater chance of developing SPM, their prognosis is limited by SPM, not by the diagnosis of CRC, in contrast to stage IV, where is limiting CRC, not SPM. According to the Cox proportional hazards model adjusted to gender, age at diagnosis, clinical stage, status of relapse and sidedness, the risk of death from CRC with SPM was significantly higher than that from CRC without SPM (HR: 1.24, 95% CI: 1.02–1.51, *p* = 0.029).

We did not find prone to develop secondary malignancies in rectosigmoid and rectal cancer patients treated by radiotherapy (Table 6), neither chemotherapy administration in our cohort of patients (Table 6) (patients diagnosed before and synchronously with the first CRC were not included).

**Table 6:** Relationship of radiotherapy and occurrence of second primary malignancy in patients with malignant neoplasm of rectosigmoid junction (C19) or rectum (C20).

	No SPM (N = 681) Count (%)	With SPM** (N = 52) Count (%)	*p* value
**Neoadjuvant radiotherapy**			
No	392 (93.1)	29 (6.9)	0.884
Yes	289 (92.6)	23 (7.4)	
**Adjuvant radiotherapy**			
No	594 (93.0)	45 (7.0)	0.831
Yes	87 (92.6)	7 (7.4)	
Radiotherapy*			
No	305 (93.3)	22 (6.7)	0.774
Yes	376 (92.6)	30 (7.4)	

	No SPM (N = 1047) Count (%)	With SPM** (N = 126) Count (%)	*p* value
**Neoadjuvant chemotherapy**			
No	787 (92.9)	60 (7.1)	1.000
Yes	303 (92.9)	23 (7.1)	
**Adjuvant chemotherapy**			
No	558 (92.4)	46 (7.6)	0.495
Yes	532 (93.5)	37 (6.5)	
Chemotherapy*			
No	417 (92.7)	33 (7.3)	0.815
Yes	673 (93.1)	50 (6.9)	

Relationship of chemotherapy and occurrence of second primary malignancy in patients with colorectal cancer (C18–C20).

SPM, second primary malignancy.

*Both neoadjuvant and adjuvant chemotherapy or radiotherapy were considered.

**Patients diagnosed before and synchronously with the first CRC were not included.

One patient with non-standard treatment was not considered.

## Discussion

The screening and personalized therapy of CRC leads to prolonged survival of these patients; however, it carries a higher chance for the development of SPMs. In this analysis, we have presented a large cohort of CRC patients treated in a single institution, with extended follow-up.

Different types of second primary malignancies in a particular type of tumor have been described^12^. Cancer of the lung, head and neck, and the genitourinary tract is associated with NSCLC^13^. Anal cancer has been increasingly associated with tumors of the oral cavity and pharynx, rectum and anal canal, larynx, lung and bronchus, ovary, vagina, and vulva, Kaposi's sarcoma and hematological malignancies^14^. Gastric cancer patients suffer more malignant tumors of the head and neck, esophagus, colon and rectum, bones and soft tissues, ovaries, bladder, or kidneys, as well as non-Hodgkin’s lymphoma^15^. Patients with bladder carcinoma are the most frequently diagnosed with SPMs and the most described second malignancy is lung cancer^16^. Compared to the general population, patients with CRC have a higher incidence of a second CRC^17^, as described in this study. Breast cancer is among the most common type of second primary malignancy in our cohort of CRC patients. As previously described, patients with breast cancer are at higher risk of developing colorectal cancer^18,19^, and they should be frequently screened for CRC, as well as patients with prostate cancer^20,21^ and malignant melanoma, where the high incidence of CRC after malignant melanoma was described by Caini et al.^22^. The incidence of gynecological cancer was similar in our patient cohort compared to the general Czech population, contrary to a Korean study^23^, but the total numbers are small overall.

For most cancers, the main risk period for the development of secondary malignancies is during the 3 years after initial diagnosis of the first tumor^24^. The highest risk of SPMs after Hodgkin´s lymphoma treatment is at 5–10 years^25^, but in solid tumors this period has not been well described. In a large Swiss study, 40% of patients developed SPMs between 1 and 5 years after the first cancer, and approximately one-third of them were diagnosed 5–10 years later^12^. In our analysis, the median time period to develop CRC after breast cancer diagnosis was 8.9 years and 3.4 years after prostate cancer diagnosis. In our data set, the median time to development of subsequent tumors was 1.7–5.2 years for the most frequent malignancies, depending on the specific diagnosis. It seems that after the first cancer diagnosis, patients should be screened for at least 5–10 years for SPMs, but this period remains unclear.

The influence of sidedness of CRC on SPMs was described in Jia et al. and Liu et al.^17,26^. The difference in the incidence of SPMs between the right and left colon was supported by Broman et al.^27^. The prognosis is better for left-sided than right-sided colon cancer^28^, and if patients survive longer, the probability of SPMs is higher for the left-side of the colon. Even so, we did not find any difference between right and left colon cancer. The high incidence of SPMs in colon cancer is in contrast with rectal cancer. According to our results, patients with rectal cancer are less likely to develop SPM than patients with right colon. This is probably not related to survival, although the prognosis of colon cancer patients is better at the early stage, but survival at advanced stages of rectal cancer is longer than colon cancer^29^.

Rectal and rectosigmoid cancer patients are often treated by radiotherapy, which has been described as a risk factor for SPMs, particularly in the pelvis, but it was not a cause of a higher incidence of SPMs in our cohort of patients^30,31^. We have not found any relationship in the occurrence of SPMs and adjuvant or neoadjuvant radiotherapy. Chemotherapy is described as a persistent risk factor for carcinogenesis^32,33^. Nevertheless, in our study, its administration was not associated with the risk of development of a SPM and patients with development of SPMs before chemotherapy/radiotherapy, and synchronously, were excluded.

Our results concord with Jia et al. which demonstrated that CRC patients with SPMs have better overall survival (OS) in the first 10 years and thereafter, have worse survival than patients without SPMs. In our study, OS was better in the first 6 years for CRC patients with SPMs, and thereafter was worse than in CRC patients without SPM. Nevertheless, the relationship between the year from CRC diagnosis and the occurrence of SPM was not statistically significant according to the logistic model (*p* = 0.306). The addition of an adjustment for the year of diagnosis to the Cox regression model also did not show significant changes in the results. An explanation of cross curve in survival analysis can also be found in the prognosis of the underlying CRC disease. Patients with a better prognosis have a higher probability of SPMs than patients with a worse prognosis, but finding the differences in OS of these patients require longer follow-up. Recently, an online competing-risk nomogram was released: Predicting Probabilities of Developing a Second Primary Malignancy for Colorectal Cancer Patients (http://biostat.fudan.edu.cn/crc)^26^.

An inherent limitation of this study is related to its retrospective nature, which is similar to all other studies dealing with this issue. The same reason limits availability of some other data which may be related to the risk of a SPM such as obesity, which increases the risk of malignancy^34^ as well as information on smoking, alcohol use, diet, sports activity and lifestyle^35,36^, which have a significant impact on cancer development, and data about these were not available for the majority of our patient cohort.

The strengths of our study include the use of a large population-based cohort of CRC patients, the patients' characteristics and treatment, detailed information on the incidence of SPMs in CRC patients from source documentation, review of medical charts, and long follow-up.

Previous studies have indicated no effect of more frequent specialized follow-up on the survival of CRC patients, but for some patients their prognosis could be limited by the occurrence of SPMs. The screening of cancer patients for the most frequent malignancies and their systematic education about risk reduction strategies should be standard in surveillance for all cancer patients, not just colorectal cancer patients.

We realize that the results from this analysis should be interpreted with caution and further studies in other centers are needed to confirm our outcomes. Understanding the risk of patients with a history of colorectal cancer would help to identify appropriate prevention strategies. Early detection of a second primary tumor should be the focus of healthcare providers as well as health insurance companies. It is imperative that professionals note that 20% of all cancer patients develop during their lives second primary tumors^37^.

## Conclusion

Patients with a diagnosis of breast cancer, prostate cancer, kidney cancer, or melanoma should be regularly screened for CRC. As well, colorectal cancer patients should also be screened for additional cancers, namely colon, breast, prostate, kidney, and bladder cancer. We recommend that CRC patients in the early stages should be screened for second primary malignancies more often than the standard population, the duration of the screening should be at least 5–10 years though intervals remain unclear. Inexpensive and noninvasive methods should be used for early detection of the most frequent SPMs. Using standard screening methods for the general population (colonoscopy or fecal occult blood test, mammography, low-dose CT of the chest under certain conditions), enriched with abdominal ultrasound, and clinical examination, we can detect the early-stage of a secondary malignancy and hopefully prolong the overall survival of CRC patients.

The early detection of cancer, whether primary or second primary, leads to lives being saved as well as economic cost savings for healthcare systems. Our goal, as professionals in healthcare is to create a screening process for SPMs that will identify the most frequent primary tumors and will be focused on the most frequent second primary malignancies bound to specific tumors, and which can prolong survival of not only colorectal cancer patients, but all cancer patients.

## Data Availability

The datasets generated and analysed during the current study are available from the corresponding author on reasonable request.

## References

[1] Siegel RL (2020). Colorectal cancer statistics, 2020. CA Cancer J. Clin..

[2] Liu L (2011). Prevalence of multiple malignancies in the Netherlands in 2007. Int. J. Cancer.

[3] Dusek L (2015). Estimating cancer incidence, prevalence, and the number of cancer patients treated with antitumor therapy in 2015 and 2020—Analysis of the Czech National Cancer Registry. Klin. Onkol..

[4] Soerjomataram I, Coebergh JW (2009). Epidemiology of multiple primary cancers. Methods Mol. Biol..

[5] Koubková L, Hrstka R, Dobes P, Vojtesek B, Vyzula R (2014). Second primary cancers—Causes, incidence and the future. Klin. Onkol..

[6] Anand P (2008). Cancer is a preventable disease that requires major lifestyle changes. Pharm. Res..

[7] Chen Q (2018). Do patients with second primary colorectal cancer hold the similar prognosis and therapeutic benefits as those with initial primary colorectal cancer?. Biomed. Res. Int..

[8] Vogt A (2017). Multiple primary tumours: Challenges and approaches, a review. ESMO Open.

[9] SEER Training Modules, Multiple primary neoplasms. *U. S. National Institutes of Health, National Cancer Institute.* Cit 15.5.2020. https://training.seer.cancer.gov/.

[10] Working Group Report (2005). International rules for multiple primary cancers (ICD-0 third edition). Eur. J. Cancer Prev..

[11] Institute of Health Information and Statistics of the Czech Republic. *National Health Information System (NHIS), Czech National Cancer Registry (CNCR).* http://www.uzis.cz/en/czech-nationalcancer-registry-cncr.

[12] Feller A (2020). The relative risk of second primary cancers in Switzerland: A population-based retrospective cohort study. BMC Cancer.

[13] Duchateau CS, Stokkel MP (2005). Second primary tumors involving non-small cell lung cancer: Prevalence and its influence on survival. Chest.

[14] Shah BK, Budhathoki N (2015). Second primary malignancy in anal carcinoma—A US population-based study. Anticancer Res..

[15] Chen SC (2016). Second primary malignancy risk among patients with gastric cancer: A nationwide population-based study in Taiwan. Gastric Cancer.

[16] Donin N (2016). Risk of second primary malignancies among cancer survivors in the United States, 1992 through 2008. Cancer.

[17] Liu L (2013). Second primary cancers in subsites of colon and rectum in patients with previous colorectal cancer. Dis. Colon Rectum..

[18] Soerjomataram I (2005). Primary malignancy after primary female breast cancer in the south of the Netherlands, 1972–2001. Breast Cancer Res. Treat..

[19] La Francis IE, Cooper RB (1992). Second primary malignancies associated with primary female breast cancer: A review of the Danbury Hospital experience. Conn. Med..

[20] Saltus CW (2019). Incidence of second primary malignancies in patients with castration-resistant prostate cancer: An observational retrospective cohort study in the United States. Prostate Cancer..

[21] Chattopadhyay S (2018). Prostate cancer survivors: Risk and mortality in second primary cancers. Cancer Med..

[22] Caini S (2014). The risk of developing a second primary cancer in melanoma patients: A comprehensive review of the literature and meta-analysis. J. Dermatol. Sci..

[23] Shin DW (2018). Secondary breast, ovarian, and uterine cancers after colorectal cancer: A nationwide population-based cohort study in Korea. Dis. Colon Rectum..

[24] Rasmussen LA (2019). Time from incident primary cancer until recurrence or second primary cancer: Risk factors and impact in general practice. Eur. J. Cancer Care (Engl.).

[25] Schaapveld M (2015). Second cancer risk up to 40 years after treatment for Hodgkin's lymphoma. N. Engl. J. Med..

[26] Jia H, Li Q, Yuan J, Sun X, Wu Z (2020). Second primary malignancies in patients with colorectal cancer: A population-based analysis. Oncologist.

[27] Broman KK, Bailey CE, Parikh AA (2019). Sidedness of colorectal cancer impacts risk of second primary gastrointestinal malignancy. Ann. Surg. Oncol..

[28] Petrelli F (2017). Prognostic survival associated with left-sided vs right-sided colon cancer: A systematic review and meta-analysis. JAMA Oncol..

[29] Lee YC, Lee YL, Chuang JP, Lee JC (2013). Differences in survival between colon and rectal cancer from SEER data. PLoS ONE.

[30] Warschkow R (2017). Secondary malignancies after rectal cancer resection with and without radiation therapy: A propensity-adjusted, population-based SEER analysis. Radiother. Oncol..

[31] Dracham CB, Shankar A, Madan R (2018). Radiation induced secondary malignancies: A review article. Radiat. Oncol. J..

[32] Liang F, Zhang S, Xue H, Chen Q (2017). Risk of second primary cancers in cancer patients treated with cisplatin: A systematic review and meta-analysis of randomized studies. BMC Cancer.

[33] Boffetta P, Kaldor JM (1994). Secondary malignancies following cancer chemotherapy. Acta Oncol..

[34] Gibson TM (2014). Body mass index and risk of second obesity-associated cancers after colorectal cancer: A pooled analysis of prospective cohort studies. J. Clin. Oncol..

[35] Morais S (2019). Second primary cancers and survival in patients with gastric cancer: Association with prediagnosis lifestyles. Eur. J. Cancer Prev..

[36] Wood ME (2012). Second malignant neoplasms: Assessment and strategies for risk reduction. J. Clin. Oncol..

[37] Berrington de Gonzalez A (2011). Proportion of second cancers attributable to radiotherapy treatment in adults: A cohort study in the US SEER cancer registries. Lancet Oncol..

